# Early Phase Enamel Bond Performance of a Two-step Adhesive Containing a Primer Derived from a Universal Adhesive

**DOI:** 10.3290/j.jad.b3559035

**Published:** 2022-11-08

**Authors:** Kei Iwase, Toshiki Takamizawa, Keiichi Sai, Sho Shibasaki, Wayne W. Barkmeier, Mark A. Latta, Atsushi Kamimoto, Masashi Miyazaki

**Affiliations:** a Graduate Student, Department of Operative Dentistry, Nihon University School of Dentistry, Tokyo Japan. Performed experiments (bond strength test), statistical evaluations, and contributed to discussion.; b Associate Professor, Department of Operative Dentistry, Nihon University School of Dentistry, Tokyo Japan. Idea, hypothesis, experimental design, wrote the manuscript, and discussed the results at all stages.; c Postdoctoral Fellow, Department of Operative Dentistry, Nihon University School of Dentistry, Tokyo Japan. Performed experiments (bond strength test), statistical evaluations, and contributed to discussion.; d Assistant Professor, Department of Orthodontics, Nihon University School of Dentistry, Tokyo, Japan. Performed experiments (specimen preparation) and contributed to discussion.; e Adjunct Professor and Dean Emeritus, Department of General Dentistry, Creighton University School of Dentistry, Omaha, NE, USA. Proofread the manuscript, contributed to discussion.; f Professor and Dean Emeritus, Department of General Dentistry, Creighton University School of Dentistry, Omaha, NE, USA. Proofread the manuscript, contributed to discussion.; g Associate Professor, Department of Comprehensive Dentistry and Clinical Education, Nihon University School of Dentistry, Tokyo Japan. Consulted on statistical evaluation, contributed to discussion.; h Professor and Chair, Department of Operative Dentistry, Nihon University School of Dentistry, Tokyo Japan. Discussed the results and commented on the manuscript at all stages.

**Keywords:** enamel bond strength, universal-adhesive-derived primer, two-step adhesive, early bonding performance.

## Abstract

**Purpose:**

To investigate the changes in the enamel bond performance of a two-step adhesive containing a primer derived from a universal adhesive in the early phase before 24 h and compare them to those of other adhesives. The Knoop hardness number (KHN) of the cured adhesive layers and resin composite was measured.

**Materials and Methods:**

A new two-step adhesive using universal adhesive technology, G2-Bond Universal, was tested. Two conventional two-step adhesives, Clearfil SE Bond 2 and OptiBond eXTRa, and an established universal adhesive, Scotchbond Universal Plus Adhesive, were used as comparison materials. Twelve specimens per group were used to measure the shear bond strength (SBS) to bovine enamel in different etching modes. The bonded specimens were stored in distilled water at 37°C for 5 min or 1, 6, 12, or 24 h before SBS testing. The KHN of the adhesive layer and resin composite was determined after the same storage periods as for SBS testing.

**Results:**

All adhesives exhibited increased SBS with prolonged storage periods, irrespective of the etching mode. The KHN of the adhesive layer and resin composite increased over time.

**Conclusions:**

There were strong positive correlations between the SBS and KHN of the adhesive layer and resin composite. Phosphoric acid pre-etching of enamel effectively increases enamel bond performance. The two-step adhesive G2-Bond Universal demonstrated significantly higher bond strength in the early phase than the other adhesives in self-etch mode.

Almost a decade ago, universal adhesives, classified as single-step self-etch adhesives, were introduced, and they are used extensively today.^3,18^ Universal adhesives have some advantages, such as simplified bonding procedures and easy handling, and they are versatile due to their multifunctional properties.^3,18^ Universal adhesives can be used in either etch-and-rinse (ER) mode or self-etch (SE) mode,^27,29^ but can also be applied as a primer for resin luting cement.^12^ Furthermore, recent universal adhesives have been reported to perform acceptably when used in abbreviated bonding procedures, eg, by reducing the application time before light irradiation of the adhesive.^21,22^ These benefits of universal adhesives are due to new technologies, such as new monomers, chemical intiators, optimization of composition, etc. However, compared with conventional two-step SE adhesives, previous studies have reported lower bonding effectiveness for universal adhesives.^26,28,30^ Although the enamel bond strength of universal adhesives in ER mode is thought to be almost equivalent to that of two-step SE adhesives with phosphoric acid pre-etching, universal adhesives in SE mode have shown significantly lower immediate enamel bond strength and bond durability than two-step SE adhesives.^27^ In addition, the bond durability of universal adhesives to intact enamel is lower than that to ground enamel, in addition to being lower than that of a two-step self-etch adhesive.^25^ Therefore, progress in developing the next generation of dental adhesives may be sought in the further improvement of universal adhesives or in utilizing universal adhesives as components of multi-step adhesives.

A new two-step adhesive, G2 Bond Universal (GC; Tokyo, Japan), has been recently developed using universal adhesive technology.^15,33^ Prior to G2 Bond Universal, the manufacturer launched a HEMA-free single-step universal adhesive (G-Premio Bond). The primer of G2 Bond Universal is similar to that of G-Premio Bond. This new adhesive contains a 2-hydroxyethyl methacrylate (HEMA)-free primer, very similar to that of G-Premio Bond, and functional monomers. It also contains a hydrophobic bonding agent which lacks both HEMA and functional monomers. Studies have reported that this adhesive exhibits equal or superior enamel and dentin bond durability compared with two conventional two-step SE adhesives after long-term water storage, thermal and fatigue stress.^15,33,35,40^

Internal and external forces are generated immediately after light irradiation of a resin composite that may diminish the bonding performance of adhesives.^5,10,18^ One of these forces is polymerization shrinkage of resin composites after setting, which generates contraction stress in the vicinity of the resin-tooth interface. In contrast, external stresses are generated during the placement of resin composite restorations, including finishing and polishing procedures. These internal and external stresses may create gaps between the resin composite and adherent surfaces, causing postoperative sensitivity and affecting the long-term stability of restorations.^10^ Although most previous studies that used bond strength tests to evaluate immediate bond effectiveness reported values measured 24 h after preparing bonded specimens, only a few studies have examined bond effectiveness before 24 h. Therefore, it may be relevant to examine the early stage of bonding performance in the first 24 h after bonding procedures, including the new two-step adhesive “G2-Bond Universal” and comparing it to different adhesives.

The purpose of this study was to determine the changes in enamel bond strength of the new type of two-step adhesive, G2-Bond Universal, in the early phase between 5 min and 24 h after specimen preparation and compare them with those of other adhesives, such as an established universal adhesive and conventional two-step self-etch adhesives. The relationship between post-polymerization effects on the cured adhesive layer, the resin composite, and enamel bond strength over time was also examined. The null hypotheses tested were: (1) the early-phase enamel bond effectiveness of G2-Bond Universal does not change during the test period; and (2) the microhardness of the adhesive layer of this new two-step adhesive does not correlate with the enamel bond strength.

## MATERIALS AND METHODS

### Study Materials

Table 1 shows the adhesives and resin composite used in this study. An adhesive containing a primer derived from a universal adhesive, G2-Bond Universal (GU, GC; Tokyo, Japan), was tested. Two representative commercially available two-step adhesives, Clearfil SE Bond 2 (CS, Kuraray Noritake; Tokyo, Japan) and OptiBond eXTRa (OX, Kerr; Orange, CA, USA), as well as an established universal adhesive, Scotchbond Universal Plus Adhesive (SP, 3M Oral Care; St Paul, MN, USA), were used as comparisons. A 35% phosphoric acid etching agent (Ultra-Etch, Ultradent; South Jordan, UT, USA) and the resin composite Clearfil AP-X (Kuraray Noritake) were used. An LED curing unit (Valo; Ultradent) was used (10-mm internal tip diameter) at a light irradiance >1000 mW/cm^2^ (standard mode), which was checked with a dental radiometer (Bluephase Meter II, Ivoclar Vivadent; Schaan, Liechtenstein).

**Table 1:** Materials used in this study

Code	Adhesive (Lot No.)	Main components	pH (primer)	Manufacturer
GB	Two-step adhesive G2-Bond Universal (Primer: 190711) (Adhesive: 190711)	Primer: 4-MET, 10-MDP, MDTP, dimethacrylate monomer, acetone, water, photoinitiator, filler Adhesive: dimethacrylate monomer, bis-GMA, filler, photoinitiator	1.5	GC; Tokyo, Japan
CS	Two-step adhesive Clearfil SE Bond 2 (Primer: 5852494) (Adhesive: 5847004)	Primer: 10-MDP, HEMA (20%–40%), water, initiators Adhesive: MDP, HEMA (20%–40%), bis-GMA (25%–45%), initiators, microfiller	2.0	Kuraray Noritake; Tokyo, Japan
OX	Two-step adhesive OptiBond eXTRa Universal (Primer: 58470004) (Adhesive: 5852494)	Primer: GPDM (20%–40%), HEMA (10%–20%), acetone (20%–40%), ethyl alcohol (1%–20%) Adhesive: GPDM (1%–10%), HEMA (10%–20%), glycerol dimethacrylate (1%–10%), ethyl alcohol (20%–40%), sodium hexafluorosilicate (<5%)	1.6	Kerr; Orange, CA, USA
SP	Universal adhesive Scothchbond Universal Plus (Adhesive: 7279357)	10-MDP, HEMA (15%–25%), Vitrebond copolymer (<2%), dimethacrylate resins (BPA derivative-free), ethanol (5%–15%), water (5%–15%), initiators, dual-cure accelerator, optimized mixture of silane, filler	2.7	3M Oral Care; St Paul, MN, USA
Resin composite		Main components		Manufacturer
Clearfil AP-X (N416713)		Bis-GMA, TEG-DMA, silane barium glass filler, silane silica filler, silanated colloidal silica, CQ, pigments, others		Kuraray Noritake

4-MET: 4-methacryloyloxyethyl trimellitate; MDP: 10-methacryloyloxydecyl dihydrogen phosphate; MDTP: 10-methacryloyloxydecyl dihydrogen thiophosphate; bis-GMA: 2,2-bis[4-(2-hydroxy-3-methacryloyloxypropoxy) phenyl] propane; HEMA: 2-hydroxyethyl methacrylate; GPDM: glycerol dimethacrylate dihydrogen phosphate; BPA: bisphenol A; TEG-DMA: triethyleneglycol dimethacrylate; CQ: dl-camphorquinone.

### Specimen Preparation

Bovine mandibular incisor enamel was used in this study. After cutting off the tooth roots using a diamond-impregnated disk in a precision sectioning saw (IsoMet 1000, Buehler; Lake Bluff, IL, USA), the central area of the labial surface was ground and polished with a grinder/polisher (Ecomet 4 Grinder Polisher, Buehler) and a wet 180-grit silicon carbide (SiC) paper (Fuji Star Type DDC; Saitama, Japan) for 5 s to create a flat enamel surface measuring approximately 8 mm in diameter. The prepared tooth was mounted in self-curing acrylic resin (Tray Resin II, Shofu; Kyoto, Japan) to expose the flattened enamel area. The enamel bonding surfaces were polished with 240- and 320-grit SiC papers (Fuji Star Type DDC) under running water for approximatel 10 s per SiC paper.^8^

### Adhesive Application Protocols

Table 2 presents the adhesive application protocols. A total of 12 specimens were used for each test group to measure the shear bond strength (SBS) to enamel in SE mode (without phosphoric acid pre-etching) or ER mode (phosphoric acid pre-etching for 15 s). SP adhesive as well as the primer and bonding agent of the two-step adhesives were applied to the enamel surfaces according to the manufacturer’s instructions (Table 2), followed by exposure to light irradiation for 10 s.

**Table 2:** Application protocols for pre-etching and the tested adhesives

Etching mode	Pre-etching protocol
SE mode (self-etch)	Phosphoric acid pre-etching was not performed.
ER mode (etch-and-rinse)	Enamel surfaces were phosphoric acid etched for 15 s. The etched surface was rinsed with water for 15 s and air dried.
Adhesive	Adhesive application protocol
GB	Primer was applied to the air-dried enamel surface for 10 s and then a strong stream of air was applied over the primer for 5 s. Bonding agent was then applied to the primed surface and was gently air thinned for 5 s. Light irradiation was performed for 10 s.
CS	Primer was applied to the air-dried enamel surface for 20 s followed by medium air pressure for 5 s. Bonding agent was then applied to the primed surface and was gently air thinned for 5 s. Light irradiation was performed for 10 s.
OX	Primer was applied to the air-dried enamel surface with rubbing action for 20 s. Medium air pressure was applied to the surface for 5 s. Bonding agent was applied to the primed surface with rubbing action for 15 s and then gently air thinned for 5 s. Light irradiation was performed for 10 s.
SP	Adhesive was applied to the air-dried enamel surface with rubbing motion for 20 s, then medium air pressure was applied to surface for 5 s. Light irradiation was performed for 10 s.

### SBS Testing

Following adhesive application, the specimens were clamped in the Ultradent Bonding Jig (Ultradent). The resin composites were placed on the enamel surfaces using a Bonding Mold Insert (2.38-mm internal diameter and 2.0-mm height), followed by light irradiation for 30 s. The molds were removed, and the bonded specimens were stored in distilled water at 37°C for 5 min or 1, 6, 12, or 24 h before SBS testing.^8,42^

After each storage period, the enamel SBS was measured using the notched-edge SBS test according to the ISO 29022 specification.^13^ The Ultradent Bonding Assembly (Ultradent) was used for determining the SBS. The specimens were loaded to failure at a 1.0 mm/min crosshead speed with the Ultradent Shearing Fixture using a universal testing machine (Type 5500R, Instron; Norwood, MA, USA). The SBSs (MPa) were calculated as the peak load at failure divided by the bonded surface area.

After testing, the bonded enamel surfaces and the debonded resin composite cylinders were observed under an optical microscope (SZH-131, Olympus; Tokyo, Japan) at 10X magnification to evaluate the failure mode. The failure modes were classified based on the substrate percentage area of the debonded specimen. If >80% of the adherent area was occupied by the adhesive, resin composite, or enamel, the failure mode was specified as 1) adhesive failure, 2) cohesive failure in resin, or 3) cohesive failure in enamel, respectively. Other failure patterns, such as partially adhesive and partially cohesive, were classified as 4) mixed failure.

### Knoop Hardness Number (KHN) of the Tested Adhesives and Resin Composite

The KHN test was conducted to examine the changes in microhardness of the adhesives and the resin composite in the early phase between 5 min and 24 h after specimen preparation, as for the SBS test. For KHN testing of the cured adhesive layer, six flat enamel specimens for each group were prepared in SE mode alone, as was done for the bond strength test. A piece of adhesive tape with a hole measuring 6 mm in internal diameter and 300 μm in thickness was attached to the enamel surface to define the area of bonding and the thickness of the adhesive layer.^42^ The adhesives were applied according to the manufacturers’ instructions (Table 2). ER mode was omitted because the influence of different etching modes on KHN was expected to be negligible, given that the adhesive layer for testing was thicker than the adhesive layer used in a clinical setting.^15,32,33^ The applied adhesive layer was covered with transparent matrix tape (Matrix Tape and Dispenser, 3M Oral Care) and then light irradiated for 10 s. After removing the matrix tape from the specimen, the adhesive surface was wiped with alcohol-saturated cotton to remove the oxygen-inhibition layer and enable determination of the KHN of the top surface of the adhesive layer. Subsequently, the specimens were stored under dark conditions in a 100%-humidity environment at 37°C for 5 min or 1, 6, 12, or 24 h before KHN testing. The KHN was obtained from the indentation after applying a 98.07-mN load for 5 s using a microhardness tester (HMV-2, Shimadzu; Kyoto, Japan). Three measurements per specimen were performed in different locations, and the mean values were calculated for the KHN value of the specimen.

For KHN testing of the cured resin composite, the resin composite paste was placed into a polytetrafluoroethylene cylindrical mold (10 mm in diameter and 2 mm in height) and covered with transparent matrix tape (Matrix Tape and Dispenser, 3M Oral Care). The specimens were light irradiated for 20 s. Immediately, the bottom surface of each specimen was manually polished using a sequence of SiC papers of up to 2000 grit (Fuji Star type DDC); the polishing time for each SiC paper was approximately 20 s. Six flat specimens were prepared and stored under dark conditions in a 100%-humidity environment at 37°C for 5 min or 1, 6, 12, or 24 h before KHN testing. The KHN was measured from the indentation after applying a 1.961-N load for 15 s time using a microhardness tester (HMV-2, Shimadzu).^34^ Three measurements per specimen were performed at different locations, and the means were calculated for each specimen.

### Scanning Electron Microscopy (SEM)

SEM (ERA-8800FE, Elionix; Tokyo, Japan) was used to observe representative adhesive-treated enamel surfaces, resin-enamel interfaces, and fracture sites on the resin side after SBS testing. Adhesive-treated enamel surfaces were prepared according to the manufacturers’ instructions (Table 2), and the uncured adhesive layer was removed by three alternating rinses using acetone and water. Specimens ground with wet #320-grit SiC paper with or without phosphoric acid etching were also made to serve as baseline.

To observe the resin-enamel interface, the bonded specimens were embedded in epoxy resin (Epon 812, Nisshin EM; Tokyo, Japan) and then longitudinally sectioned using a low-speed saw (IsoMet 1000). The sectioned surfaces were mirror-polished to achieve a high gloss with abrasive disks (Fuji Star Type DDC), followed by a sequence of diamond pastes of 6.0-, 3.0-, 1.0-, and 0.25-μm particle size (DP-Paste, Struers; Ballerup, Denmark). The polishing time for each SiC paper and diamond paste was approximately 3 min. Debonded resin composite cylinders were ultrasonically cleaned for 30 s and subsequently air dried. Apart from debonded specimens, all the SEM specimens were dehydrated in ascending grades of tert-butyl alcohol and then transferred from the final 100% bath to a freeze dryer (Model ID-3; Elionix) for 30 min. The specimens of the resin-enamel interfaces were then subjected to argon-ion beam etching (EIS-200ER, Elionix) for 40 s, with the ion beam (accelerating voltage 1.0 kV, ion current density 0.4 mA/cm^2^) directed perpendicular to the polished surfaces.^8^ Finally, all the SEM specimens were coated in a vacuum evaporator (Quick Coater, Type SC-701, Sanyu Electron; Tokyo, Japan) with a thin film of gold to increase the conductivity of the specimens. The SEM observations were performed at an operating voltage of 10 kV.

### Statistical Analysis

Sample sizes for the SBS test and KHN measurement were calculated based on our previous study^42^ using statistical software (Sigma Plot v 13, Systat Software, SPSS; Chicago, IL, USA). The experiments were conducted using 12 specimens for the SBS test and 6 specimens for KHN measurements of the cured adhesive layer and resin composite.

The SBS and KHN data for each group were tested for homogeneity of variance (Bartlett’s test) and normal distribution (Kolmogorov-Smirnov test). The SBS data were subjected to three-way ANOVA followed by Tukey’s HSD test (α = 0.05). The factors analyzed were etching mode, storage time, and adhesive. One-way ANOVA followed by Tukey’s HSD test (α = 0.05) were employed for comparisons within subsets of data.

The KHN data of the cured adhesive layer were subjected to two-way ANOVA followed by Tukey’s HSD test (α = 0.05). The factors analyzed were storage time and adhesive. The KHN data of the cured resin composite were also analyzed using one-way ANOVA followed by Tukey’s HSD test (α = 0.05). Furthermore, linear regression analysis for each adhesive explored the relationship between the SBS and KHN of the adhesive layer or resin composite over time. All statistical analyses were performed using the statistical software Sigma Plot (v 13).

## RESULTS

### SBS

**Fig 1:**
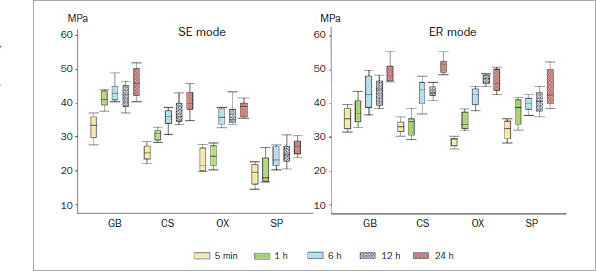
Early-phase enamel bond strength with different etching modes. GB: G2-Bond Universal, CS: Clearfil SE Bond, OX: OptiBond eXTRa, SP: Scotchbond Universal Plus, SE: self-etching, ER: etch-and-rinse.

Tables 3 and 4 and Fig 1 present the mean SBS results. The results of three-way ANOVA revealed that the etching mode, storage period, and adhesive significantly impacted the enamel mean SBS (p < 0.001). All pairwise interactions between the factors were significant (p < 0.05), and the three-way interaction among the etching mode, storage period, and adhesive was also significant (p < 0.001).

**Table 3:** Enamel bond strengths of the adhesives over time in SE mode

	5 min	1 h	6 h	12 h	24 h
GB	32.8 (3.3)^aC^ [71.1%]	41.3 (2.3)^aB^ [89.6%]	43.4 (2.9)^aAB^ [94.1%]	42.0 (3.4)^aB^ [91.1%]	46.1 (4.1)^aA^ [100%]
CS	25.3 (2.2)^bD^ [62.6%]	30.6 (1.6)^bC^ [75.7%]	35.6 (2.6)^bB^ [88.1%]	37.3 (3.3)^bAB^ [92.3%]	40.4 (3.6)^bA^ [100%]
OX	23.1 (3.3)^bB^ [60.0%]	24.4 (2.9)^cB^ [63.4%]	35.9 (2.3)^bA^ [93.2%]	36.6 (3.4)^bA^ [95.1%]	38.5 (2.2)^bA^ [100%]
SP	18.9 (3.1)^cB^ [69.7%]	20.0 (4.0)^dB^ [73.8%]	24.1 (2.9)^cA^ [88.9%]	25.0 (3.3)cA [92.3%]	27.1 (2.3)^cA^ [100%]

Percentages in brackets indicate bond strength relative to 24 h strength (n = 12), mean (SD) in MPa. Same lowercase letter in columns indicates no significant difference (p < 0.05). Same capital letter in rows indicates no significant difference (p < 0.05).

**Fig 2:**
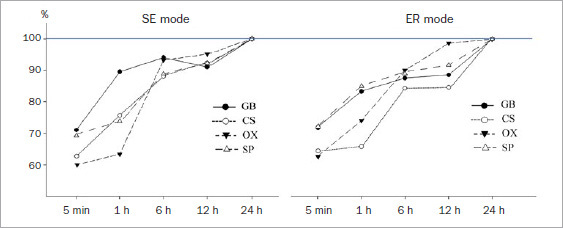
Changes in shear bond strength (%) with different etching modes. GB: G2-Bond Universal, CS: Clearfil SE Bond, OX: OptiBond eXTRa, SP: Scotchbond Universal Plus, SE: self-etching, ER: etch-and-rinse.

All the adhesives demonstrated increased mean SBS with prolonged storage periods in both etching modes. Defining the mean adhesive SBS as 100% at 24 h in SE mode, the mean SBS (5-min and 1-, 6-, and 12-h groups) ranged from 71.1% to 94.1% in GB, 62.6% to 92.3% in CS, 60.0% to 95.1% in OX, and 69.7% to 92.3% in SP (Table 3 and Fig 2). Although all the adhesives in the 5-min and 1-h groups exhibited significantly lower mean SBS than those in the 24-h groups, GB showed somewhat higher percentages of the final bond strength in the 5-min and 1-h groups than did the other adhesives. However, OX showed the lowest percentages at 5 min and 1 h of all adhesives tested. Comparing mean SBS at the same storage period between different adhesives revealed that GB had significantly higher mean SBS than did the other adhesives in every storage period. In contrast, SP showed significantly lower mean SBS than did the other adhesives at each storage period.

When the mean SBS of the tested adhesive was defined as 100% at 24 h in ER mode, the SBS (5-min and 1-, 6-, and 12-h groups) ranged from 72.0% to 88.6% in GB, 64.5% to 84.6% in CS, 62.7% to 98.7% in OX, and 72.3% to 91.7% in SP (Table 4 and Fig 2). The rate of increase in ER mode showed a trend similar to that in SE mode, that is, a gradual increase over time. All the adhesives showed significantly lower mean SBS in the 5-min and 1- and 6-h groups than those in the 24-h groups. GB and SP exhibited somewhat higher percentages in the 5-min and 1-h groups than did the other adhesives. A comparison of mean SBS at the same storage period between different adhesives revealed that, although GB showed significantly higher mean SBS in the 5-min and 1-h groups than the other two-step adhesives, there were no significant differences in mean among the two-step adhesives at the other storage periods.

**Table 4:** Enamel bond strengths of the adhesives over time in ER mode

	5 min	1 h	6 h	12 h	24 h
GB	35.5 (3.0)^aC^ [72.0%]	41.1 (3.3)^aB^ [83.4%]	43.2 (4.8)^aB^ [87.6%]	43.7 (3.8)^abB^ [88.6%]	*49.3 (3.1)^abA^ [100%]
CS	*33.1 (1.9)^bC^ [64.5%]	*33.8 (3.2)^cC^ [65.9%]	*43.3 (3.9)^aB^ [84.4%]	*43.4 (1.7)^abB^ [84.6%]	*51.3 (2.3)^aA^ [100%]
OX	*29.3 (1.3)^cD^ [62.7%]	*34.6 (2.5)^bcC^ [74.1%]	*42.1 (2.6)^aB^ [90.1%]	*46.1 (1.3)^aA^ [98.7%]	*46.7 (3.2)^bcA^ [100%]
SP	*32.1 (2.7)^bC^ [72.3%]	*37.8 (3.7)^abB^ [85.1%]	*39.8 (2.1)^aB^ [89.6%]	*40.7 (3.1)^bAB^ [91.7%]	*44.4 (5.2)^cA^ [100%]

Percentages in brackets indicate bond strength relative to 24 h strength (n = 12), mean (SD) in MPa. Same lowercase letter in columns indicates no significant difference (p < 0.05). Same capital letter in rows indicates no significant difference (p < 0.05). Asterisk indicates significant differences between SE mode and ER mode (p < 0.05).

A comparison of mean SBS in the SE and ER modes for the same adhesive at the same storage period showed that CS, OX, and SP had significantly higher mean SBS in ER mode than in SE mode at every storage time. However, GB showed no significant differences in mean SBS between the ER and SE modes, apart from in the 24-h group (Tables 3 and 4, Fig 1).

### Failure Mode

**Fig 3:**
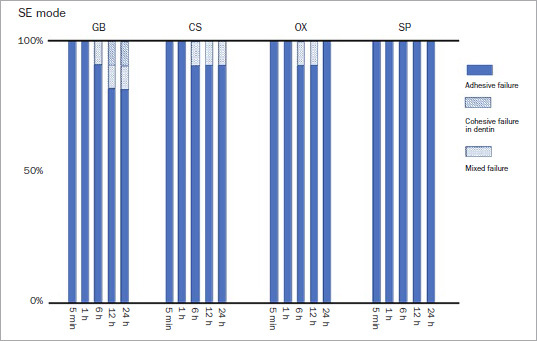
Failure mode after SBS in SE mode. GB: G2-Bond Universal, CS: Clearfil SE Bond, OX: OptiBond eXTRa, SP: Scotchbond Universal Plus.

**Fig 4:**
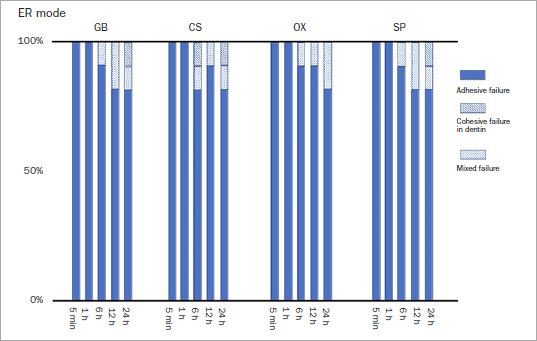
Failure mode after SBS in ER mode. GB: G2-Bond Universal, CS: Clearfil SE Bond, OX: OptiBond eXTRa, SP: Scotchbond Universal Plus.

The predominant failure mode was adhesive, irrespective of the etching mode or storage period (Figs 3 and 4). All the debonded specimens exhibited adhesive failure in the 5-min and 1-h groups regardless of etching mode or adhesive type. For all adhesives, mixed failure and cohesive failure in enamel occurred in the 6-, 12-, and 24-h groups in both etching modes, apart from SP in SE mode. Moreover, the frequencies of cohesive failure in enamel and mixed failure were higher in ER mode than in SE mode.

### KHN of the Tested Adhesives and Resin Composite

**Fig 5:**
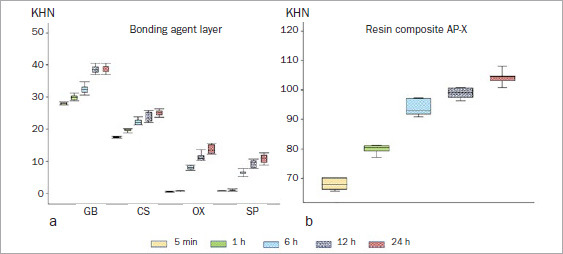
KHN in the cured adhesive layer and resin composite AP-X. a. cured adhesive layer; b. resin composite AP-X. GB: G2-Bond Universal, CS: Clearfil SE Bond, OX: OptiBond eXTRa, SP: Scotchbond Universal Plus.

Table 5 and Fig 5a show the changes in KHN of the adhesive layer over time. The results of two-way ANOVA demonstrated that the storage period and adhesive had a significant impact on the KHN of the adhesive layer (p < 0.001), and that the two-way interaction between the storage period and adhesive was also significant (p < 0.001).

**Table 5:** Changes in the KHN of the adhesive layer over time

	5 min	1 h	6 h	12 h	24 h
GB	27.9 (0.5)^aD^ [71.5%]	29.9 (0.8)^aC^ [76.7%]	32.4 (1.3)^aB^ [83.1%]	38.7 (1.3)^aA^ [99.2%]	39.0 (1.2)^aA^ [100%]
CS	17.6 (0.2)^bE^ [69.8%]	19.8 (0.5)^bD^ [78.6%]	22.3 (0.9)^bC^ [88.5%]	23.6 (1.4)^bB^ [93.7%]	25.2 (0.9)^bA^ [100%]
OX	0.5 (0.1)^cD^ [3.6%]	0.8 (0.1)^cD^ [5.8%]	8.1 (0.6)^cC^ [59.1%]	11.3 (1.2)^cB^ [82.5%]	13.7 (1.4)^cA^ [100%]
SP	0.7 (0.1)^cD^ [6.4%]	1.1 (0.2)^cD^ [10.1%]	6.4 (0.7)^dC^ [58.7%]	9.3 (1.2)^dB^ [85.3%]	10.9 (1.3)^dA^ [100%]

Percentages in brackets indicate KHN relative to 24 h value (n = 6), mean (SD). Same lowercase letter in columns indicates no significant difference (p < 0.05). Same capital letter in rows indicates no significant difference (p < 0.05).

**Fig 6:**
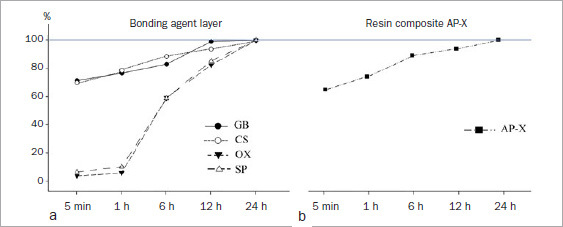
Changes in KHN (%) in the cured adhesive layer and resin composite AP-X. a. cured adhesive layer; b. resin composite AP-X. GB: G2-Bond Universal, CS: Clearfil SE Bond, OX: OptiBond eXTRa, SP: Scotchbond Universal Plus.

The KHN of the adhesive layer was dependent on both the adhesive and the storage period. However, all the adhesives exhibited increased KHN with an increased storage period. When the KHN of the adhesive layer in the 24-h storage group for each tested adhesive was defined as 100%, the KHN in the groups stored < 24 h ranged from 71.5% to 99.2% in GB, 69.8% to 93.7% in CS, 3.6% to 82.5% in OX, and 6.4% to 85.3% in SP (Table 5 and Fig 6a). Among the two-step adhesives, although GB and CS demonstrated a similar trend, the trend of OX was similar to that of the universal adhesive SP. GB and CS showed approximately 70% KHN in the groups immediately after light irradiation (5 min), but OX and SP showed <10%. GB showed significantly higher KHN than the other adhesives, irrespective of the storage time. Although CS showed significantly lower KHN than GB at all storage periods, CS showed significantly higher KHN than OX and SP. The universal adhesive SP showed significantly lower KHN than the other adhesives in the 6-, 12-, and 24-h groups.

Table 6 and Fig 5b show the changes in KHN of the resin composite over time. AP-X exhibited increased KHN with an increased storage period. There were significant differences in KHN between the storage groups. When the KHN of the resin composite in the 24-h storage group was defined as 100%, the KHN in earlier groups ranged from 65.1% to 94.8% (Fig 6b).

**Table 6:** Changes in the KHN of the resin composite over time

	5 min	1h	6h	12h	24h
KHN	67.9 (1.5)^e^ [65.1%]	80.1 (1.4)^d^ [76.8%]	93.9 (2.5)^c^ [90.0%]	98.9 (1.7)^b^ [94.8%]	104.3 (2.0)^a^ [100%]

Percentages in brackets indicate KHN values relative to values at 24 h (n = 6), mean (SD). Same lower case letter indicates no difference (p < 0.05).

### Linear Regression Analysis Between SBS and KHN Over Time

The correlation between the SBS in SE mode and KHN of the adhesive layer over time is shown in Table 7. According to the linear regression analysis, the correlation coefficient (R) for the adhesives ranged from 0.744 to 0.997. Although GB showed only a strong correlation between the SBS and KHN of the adhesive layer, the other adhesives showed extremely strong correlations between the SBS and KHN of the adhesive layer. Although the SBS of GB did not show a significant linear relationship with the KHN of the adhesive layer (p = 0.150), the SBS of the other adhesives did (p < 0.05). Over time, the overall relationship between the SBS and KHN of the adhesive layer was 0.851, which is an extremely strong correlation and statistically significant (p < 0.05).

**Table 7:** Linear regression analysis between SBS in SE mode and KHN of the adhesive layer over time

	R	R_f_^2^	SEE	p-value	Regression equations
GB	0.744	0.404	3.861	0.150	16.49 + 0.734 KHN
CS	0.997	0.991	0.574	<0.001	- 8.66 + 1.959 KHN
OX	0.975	0.935	1.865	0.005	23.53 + 1.187 KHN
SP	0.988	0.968	0.793	0.002	2.69 + 0.734 KHN
Overall	0.851	0.708	4.533	<0.001	22.80 + 0.567 KHN

R: correlation coefficient; R_f_^2^: adjusted determination coefficient; SEE: standard error of estimate. Explanatory variable: SBS; response variable: KHN.

The correlation between the SBS in SE mode and KHN of the resin composite AP-X over time is shown in Table 8. As demonstrated by the linear regression analysis, the correlation coefficient (R) for the adhesives ranged from 0.910 to 0.980, and all the adhesives exhibited an extremely strong correlation between the SBS and KHN of the resin composite. All the adhesives demonstrated a significant linear relationship between the SBS and KHN of the resin composite (p < 0.05). Over time, the overall relationship between the SBS and KHN of the resin composite was 0.573, which is a strong, statistically significant correlation (p < 0.05).

**Table 8:** Linear regression analysis between SBS in SE mode and KHN of the resin composite over time in SE mode

	R	R_f_^2^	SEE	p-value	Regression equations
GB	0.910	0.770	2.396	0.032	13.82 + 0.307 KHN
CS	0.998	0.996	0.446	<0.001	- 1.78 + 0.400 KHN
OX	0.963	0.903	1.865	0.009	- 10.24 + 0.470 KHN
SP	0.980	0.947	0.793	0.003	2.686 + 0.228 KHN
Overall	0.573	0.291	7.066	0.008	1.004 + 0.353 KHN

R: correlation coefficient; R_f_^2^: adjusted determination coefficient; SEE: standard error of estimate. Explanatory variable: SBS; response variable: KHN.

### SEM Observations

**Fig 7:**
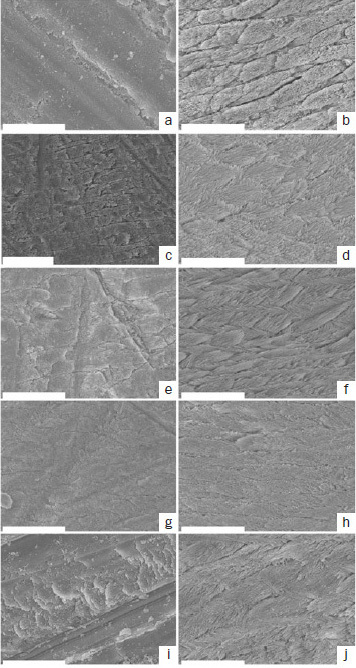
Representative SEM images of the treated enamel surface (original magnification 5000X). a. ground with SiC paper #320; b. phosphoric acid etching for 15 s; c. GB in SE mode; d. GB in ER mode; e. CS in SE mode; f. CS in ER mode; g. OX in SE mode; h. OX in ER mode; I. SP in SE mode; J. SP in ER mode.

Representative SEM images of baseline and treated enamel surfaces are depicted in Fig 7. The baseline specimen ground with SiC papers revealed scratch marks, and the surface was covered with a smear layer (Fig 7a). The smear layer was completely removed from the baseline specimen by phosphoric acid etching, which yielded a typical etching pattern (Fig 7b). For the treated enamel surfaces with different adhesives in SE mode, all the two-step adhesives exhibited a similar morphological appearance. Most of the smear layer was removed, revealing a shallow etching pattern (Figs 7c, 7e, 7g). However, the morphological appearance of SP was similar to the baseline specimen ground with SiC papers, and this adhesive showed remaining scratches and smear layer on the treated surface (Fig 7i). For the enamel surfaces treated with different adhesives in ER mode, although all adhesives had an appearance similar to that of the baseline specimen treated with phosphoric acid (Figs 7d and f), the spicular etching pattern appeared to be collapsed in OX and SP (Figs 7h and j).

**Fig 8:**
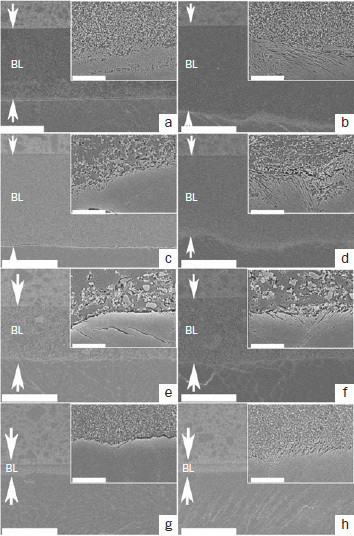
Representative SEM images of resin-enamel interfaces (original magnifications 1000X and 20,000X [insets]. a. GB in SE mode; b. GB in ER mode; c. CS in SE mode; d. CS in ER mode; e. OX in SE mode; f. OX in ER mode; g. SP in SE mode; h. SP in ER mode. BL: adhesive layer; the white arrows indicate the adhesive layer.

Figure 8 shows representative SEM images of resin-enamel interfaces. All the adhesives exhibited excellent adaptation between the adhesive layer and decalcified enamel in both etching modes. A comparison within the same etching mode revealed no obvious differences in the ultrastructure at the interface between the adhesive layer and decalcified enamel between the adhesives. The smear layer was completely dissolved, and resin tags were observed, a result of adhesive penetration into the demineralized enamel in ER mode (Figs 8b, 8d, 8f, 8h). However, such penetration was not visible in SE mode (Figs 8a, 8c, 8e, 8g). Although the thicknesses of the adhesive layer were similar between GB and CS in both etching modes, those of OX and SP were different. The adhesive layer of GB and CS was approximately 40- to 50-μm thick (Figs 8a–8d), and the adhesive layer of OX was approximately 20- to 30-μm thick (Figs 8e and 8f); whereas that of SP was approximately 5-μm thick (Figs 8g and 8h).

**Fig 9:**
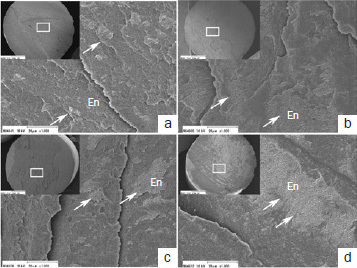
Representative debonded failure sites of GB (original magnifications 40X [insets] and 1000X). a. GB in SE mode at 5 min; b. GB in ER mode at 5 min; c. GB in SE mode at 24 h; d. GB in ER mode at 24 h. En: enamel. Arrows indicate cracks and cleavages.

**Fig 10:**
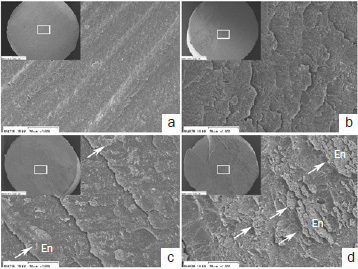
Representative debonded failure sites of OX (original magnifications 40X [insets] and 1000X). a. OX in SE mode at 5 min; b. OX in ER mode at 5 min; c. OX in SE mode at 24 h; d. OX in ER mode at 24 h. En: enamel. Arrows indicate cracks and cleavages.

**Fig 11:**
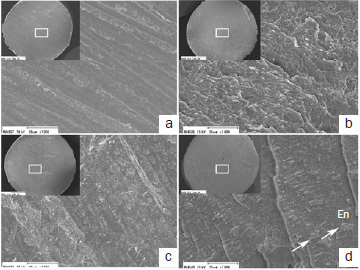
Representative debonded failure sites of SP (original magnifications 40X [insets] and 1000X). a. OX in SE mode at 5 min at magnifications of 40X and 1000X; b. OX in ER mode at 5 min; c. OX in SE mode at 24 h; d. OX in ER mode at 24 h. En: enamel. Arrows indicate cracks and cleavages.

Representative SEM images of the resin side of the debonded specimens after SBS testing are depicted in Figs 9–11. The failure patterns depended on the adhesive, etching mode, and storage time. The failure patterns of the two-step adhesives (Figs 9 and 10) tended to have several cracks and cleavages. They had more attached enamel fragments (indicated by white arrows) compared with the universal adhesive SP (Fig 11). Moreover, striation and attached enamel fragments were more clearly observed at 24-h storage in ER mode than in SE mode at 5-min storage, irrespective of the adhesive. The failure patterns at 5-min storage in SE mode were relatively flat, and beach marks were not visible in OX and SP (Figs 10a and 11a).

## DISCUSSION

Although there are conflicting opinions regarding whether bovine teeth are an appropriate substitute for human teeth in bond strength tests, previous studies showed no significant differences in shear bond strength results between bovine and human teeth.^41^ Further, it is easy to obtain many specimens of bovine teeth in good condition, with less variable composition than human teeth. Bovine teeth have large flat areas and have not had prior acid challenges that might affect the outcome. Therefore, bovine enamel was used as a substitute for human enamel in this study, as in previous studies.^8,33^

In this study, we focused on the enamel bonding efficacy of the two-step adhesive GB within 24 h of bonding, and compared it to different types of adhesives using bond strength testing and KHN of the cured adhesive layer and resin composite. The new two-step adhesive GB applies universal-adhesive technology (ie, a HEMA-free primer) and uses a hydrophobic bonding agent which does not contain HEMA, functional monomers, solvent, or water.^15,33,35^ One of the comparison materials, CS, is the gold-standard two-step SE adhesive due to its excellent laboratory and clinical performance.^17,38^ OX is the successor adhesive to the two-step self-etch adhesive OptiBond XTR, and it can be used in either ER or SE mode. SP is representative of second-generation universal adhesives and is the successor to the first commercially available single-step universal adhesive, Scotchbond Universal. SP can also be used as a primer for resin luting cement due to the optimization of the adhesive composition and a dual-cure accelerator.^1^

The SBS tests showed that the etching mode, storage period, and adhesive significantly affected the enamel SBS (p < 0.001). Moreover, all the pairwise interactions were significant (p < 0.05), and the three-way interaction among the factors was also significant (p < 0.001). Although each factor influenced each of the others, when focusing on the storage period, a prolonged storage period had a positive impact on the mean SBS regardless of the adhesive or etching mode. However, the effect appears to have been weaker for GB. ER mode also had a positive impact on SBS regardless of the adhesive or storage periods, but this effect too was much weaker for GB. These interactions point to GB being less sensitive to changes in the conditions than the other adhesives.

Comparing the same adhesive in different etching modes at the same storage period revealed that SP and the two-step adhesives other than GB had significantly higher mean SBS in ER mode than in SE mode at all storage periods. This phenomenon may be explained by different levels of etching in different etching modes and the affinity of functional monomers for enamel hydroxyapatite (HAp). A previous study demonstrated that the depth of demineralized enamel formed when using universal adhesives and a two-step SE adhesive without pre-etching was approximately 3 μm, in contrast to approximately 20 μm with phosphoric acid pre-etching.^31^ These differences in etching ability might affect the mechanical interlocking between the decalcified enamel substrate and the cured adhesive layer. Although all the tested adhesives contain functional monomers to establish chemical bonding with the decalcified enamel substrate, the affinity of functional monomers for enamel HAp is lower than that for dentin.^14^ In addition, although most adhesives in this study employ 10-methacryloyloxydecyl dihydrogen phosphate (MDP) as a functional monomer, OX employs the functional monomer glycerol phosphate dimethacrylate (GMDP), and the chemical bonding ability of GMDP is lower than that of MDP.^43^ Therefore, phosphoric acid pre-etching of enamel might still be important to enhance the enamel bonding performance of universal adhesive and two-step adhesives.

Nevertheless, excluding the 24 h group of GB, most storage groups showed no significant differences in SBS between the ER and SE modes. This might be explained by the fact that the pH (1.5) of the GB primer is lower than those of the two-step adhesives CS (2.0) and OX (1.6) as well as the universal adhesive SP (2.7). The lower pH of the GB primer might enhance enamel surface irregularities, leading to increased mechanical interlocking in SE mode. Moreover, although all the adhesives contain phosphoric acid ester functional monomers, such as 10-methacryloyloxydecyl dihydrogen phosphate (MDP) or glycerol phosphate dimethacrylate (GPDM), GB contains a carboxylic acid functional monomer. 4-methacryloyloxyethyltrimellitate acid (4-MET), in addition to MDP. These different types of functional monomers might enhance the enamel bond strength in the early phase.

In both etching modes, all the adhesives exhibited higher mean SBS after prolonged storage periods (1 h, 6 h, 12 h, and 24 h), and the mean SBS of all the adhesives at 5 min and 1 h were significantly lower than those at 24 h in both adhesion strategies. This result is consistent with previous studies investigating the early enamel and dentin bond performance of universal adhesives.^8,42^ Therefore, the first null hypothesis, that the enamel bond effectiveness in the early phase of the new type of the two-step adhesive GB would not change during the test period (5 min, 1 h, 6 h, 12 h, and 24 h), was rejected. The tendency of bond strengths to increase over time may be explained by post-polymerization effects in both the cured adhesive layer and the resin composite. Post-polymerization effects influence the mechanical properties in the vicinity of the resin-tooth interface and may cause an increase in SBS over time in the early phase before 24 h.

The surface hardness of a resin-based material might be related to its mechanical properties, abrasion resistance, and degree of conversion.^4,9,20^ To understand the mechanical properties in the vicinity of the resin-enamel interface, the KHN values of the cured adhesive layer and resin composite were measured at 5 min, 1 h, 6 h, 12 h, and 24 h after specimen preparation. The KHN of the adhesive layers of all the adhesives and the resin composite increased with time, as was the case for SBS. Therefore, the second null hypothesis, that microhardness of the cured adhesive layer of the new type of two-step adhesive GB would not show any association with enamel bond strength, was rejected.

However, the increase in KHN of the adhesive layer was different for different adhesives. The KHN test results show that the adhesives fall into 2 groups in terms of KHN changes over time: GB and CS in one group, and OX and SP in the other. For GB and CS, the KHN of the adhesive layers in the 5-min group was approximately 70% (defining the KHN of each adhesive at 24 h as 100%), and their KHN values gradually increased as the storage time increased up to 24 h. In contrast, there was a noticeable delay in increasing KHN for SP and OX. Their adhesive layers at 5 min and 1 h showed KHN values ≤10%, and those at 6 h did not reach 60%. The bonding agent components may explain the discrepant trends in different adhesives. A low-pH environment due to the acidic functional monomer and residual water or solvent in the cured adhesive layer might delay the post-polymerization effects.^24^

Although GB and CS exhibited a similar trend, GB showed a significantly higher KHN than did CS. GB may create a hydrophobic adhesive layer, as it is free of HEMA and functional monomer, but CS contains HEMA and MDP, which may explain this finding. Because it is a hydrophilic functional monomer with only one polymerization group and cannot form cross-links, HEMA has low reactivity and is not hydrolytically stable.^36,44^ Matsui et al^16^ investigated the effect of the presence or absence of 10-MDP in the adhesive on the ultimate tensile strength (UTS) of the cured adhesive, demonstrating that the MDP-free group had higher UTS than did the group with MDP, irrespective of the storage period. Therefore, compared to GB, the adhesive layers of CS exhibited significantly lower KHN values at all measurement periods.

In this study, the KHN of the resin composite at 5 min was approximately 65% of the value at 24 h and gradually increased up to 24 h. The post-polymerization effect in the resin composite is another factor that increases the mean SBS at different storage times under 24 h. A previous study examined the enamel bond effectiveness of four universal adhesives and the relationship between enamel bond strength and flexural properties of the resin composite over time between 5 min and 24 h after making specimens.^8^ The mean SBS of the universal adhesive and the flexural properties of the resin composite increased with time. Other studies also observed a strong positive correlation between the bond strength and flexural properties of resin composites.^7,11^ Although the method used to evaluate the mechanical properties of the resin composite differed from that employed in the present study, the results for KHN in this study showed the same trends as previous studies, in which flexural properties increased with time.

Overall, the present linear regression analysis showed an extremely strong correlation between SBS and KHN of the adhesive, as well as a strong correlation between the mean SBS and KHN of the cured resin composite over time. It is probable that the increased mechanical properties of the cured adhesive layer and the resin composite lead to more uniform stress distribution at the bonding interface, avoiding a concentration of stress at the point of load application.^2^ The enhanced strength and stiffness in the vicinity of the interface over time can be considered important factors for the increase in bond strength in the early phase before 24 h. When considering the bond strength tests, the bond strength may be associated with energy consumption in the plastic deformation of the resin-tooth interface when the fabricated specimens fail.^2,23^ The mechanical properties of each material that composed the adhesion interface might affect the plastic deformation in the vicinity of the interface.^39^ However, it is possible that the mechanical properties of the cured adhesive layer make a greater contribution to enamel bond performance in the testing periods before 24 h (ie, 5 min and 1 h) than does the resin composite. The correlation between the overall SBS and KHN of the adhesive layer was higher than that with the KHN of the resin composite.

In the present study, GB in SE mode and at 5 min and 1 h in ER mode showed significantly higher SBS than did the other adhesives. It can be hypothesized that the higher mechanical properties of the GB adhesive layer, due to the highly hydrophobic and somewhat thicker adhesive layer, contributed to the higher mean SBS. The adhesive layer thickness may also be considered an important factor in bond performance.^6,8,15,33,39^ SEM observations showed similar adhesive thicknesses for GB and CS, thicker adhesive layers compared to OX and SP. Previous studies investigated the effect of adhesive layer thickness on the enamel and dentin bond performance of three two-step adhesives.^15,33^ Specimens with adhesive thicknesses of approximately 50–60 μm exhibited higher SBS than did specimens with thinner adhesive layers. Better stress distribution may explain this phenomenon in the vicinity of the interface, due to the increased size of the plastic zone and improved elasticity.^6,39^ This speculation may be supported by the other results of this study, in which the universal adhesive SP showed significantly lower mean SBS than did the two-step adhesives at all time intervals. The adhesive layer of SP was very much thinner than that of the other adhesives; in addition, SP contains a relatively large quantity of water and solvents that may lead to a lower KHN of the adhesive layer and result in lower mean SBS.

In a clinical setting, external forces may be generated due to the removal of the matrix and the finishing and polishing procedures,^10,11^ in addition to internal forces following placement of resin composite restorations. Although phosphoric acid pre-etching is useful to enhance enamel bond effectiveness in both the two-step self-etch adhesives and the universal adhesive, other factors, such as the mechanical properties of interface materials and the polymerization properties of adhesives and resin composite, may be important in the early phase, as indicated by the gradual increase in mean SBS in ER mode up to 24 h.

Although we focused on the enamel bond strength of the two-step adhesive containing a universal-adhesive-derived primer up to 24 h, not enough information about the enamel bond durability of this two-step adhesive is available. Therefore, further laboratory research and clinical study are needed to investigate enamel bond durability of this new two-step adhesive.

## CONCLUSION

Although all the adhesives showed significantly lower mean SBS in the 5 min, 1 h, and 6 h groups than at 24 h, most adhesives showed significantly higher mean SBS in ER mode than in SE mode. Therefore, phosphoric acid pre-etching of enamel before applying a primer or adhesive was effective for increasing the enamel bond strength of the adhesives in the early phase (ie, 5 min, 1 h, and 6 h). The two-step adhesive using a universal-adhesive-derived primer demonstrated significantly higher enamel bond strengths in the early phase than did the other adhesives in SE mode.

## Clinical relevance:

Phosphoric acid pre-etching may be helpful to enhance enamel bond effectiveness in the early phase after placement of resin composite restorations, regardless of the adhesive. It may also be beneficial to use the new two-step adhesive containing a universal adhesive’s primer if phosphoric acid pre-etching is not performed.
